# Immunogenicity and Safety of an AS03-Adjuvanted H7N9 Pandemic Influenza Vaccine in a Randomized Trial in Healthy Adults

**DOI:** 10.1093/infdis/jiw414

**Published:** 2016-09-07

**Authors:** Anuradha Madan, Nathan Segall, Murdo Ferguson, Louise Frenette, Robin Kroll, Damien Friel, Jyoti Soni, Ping Li, Bruce L. Innis, Anne Schuind

**Affiliations:** 1 GSK, Collegeville; 2 GSK Vaccines, King of Prussia, Pennsylvania; 3 Clinical Research Atlanta, Stockbridge, Georgia; 4 Seattle Women's: Health, Research, Gynecology, University of Washington, Seattle; 5 Colchester Research Group, Truro, Nova Scotia; 6 QT Research, Sherbrooke, Quebec, Canada; 7 GSK Vaccines, Wavre, Belgium; 8 GSK Pharmaceuticals, Bangalore, India

**Keywords:** influenza, pandemic, H7N9, vaccine, AS03, adjuvant system, antigen sparing

## Abstract

**Background:**

Almost 700 cases of human infection with avian influenza A/H7N9 have been reported since 2013. Pandemic preparedness strategies include H7N9 vaccine development.

**Methods:**

We evaluated an inactivated H7N9 vaccine in an observer-blind study in healthy adults aged 18–64 years. Participants (420) were randomized to receive 1 of 4 AS03-adjuvanted vaccines (low or medium dose of hemagglutinin with AS03_A_ or AS03_B_), one nonadjuvanted vaccine, or placebo. The coprimary immunogenicity objective determined whether adjuvanted vaccines elicited an immune response against the vaccine-homologous virus, 21 days after the second vaccine dose per US and European licensure criteria in the per-protocol cohort (n = 389).

**Results:**

All adjuvanted vaccines met regulatory acceptance criteria. In groups receiving adjuvanted formulations, seroconversion rates were ≥85.7%, seroprotection rates ≥91.1%, and geometric mean titers ≥92.9% versus 23.2%, 28.6%, and 17.2 for the nonadjuvanted vaccine. The AS03 adjuvant enhanced immune response at antigen-sparing doses. Injection site pain occurred more frequently with adjuvanted vaccines (in ≤98.3% of vaccinees) than with the nonadjuvanted vaccine (40.7%) or placebo (20.0%). None of the 20 serious adverse events reported were related to vaccination.

**Conclusions:**

Two doses of AS03-adjuvanted H7N9 vaccine were well tolerated and induced a robust antibody response at antigen-sparing doses in healthy adults.

**Clinical Trials Registration:**

NCT01999842.

Periodic outbreaks of H7 avian influenza A virus infections occur in poultry worldwide, with sporadic transmission to humans. In 2003, an outbreak of H7N7 disease in The Netherlands resulted in 89 human infections and 1 death, with evidence of limited human-to-human transmission [1]. Human infections with H7N9 viruses were first reported in China in February 2013; to the present time, there have been 3 waves of infection [2]. As of December 2015, a total of 683 laboratory-confirmed cases, including 275 deaths, had been reported to the World Health Organization [2, 3]. The case fatality rate of H7N9 influenza is approximately 40% [2, 3]. The virus can cause rapidly progressive pneumonia, often complicated by extrapulmonary disease associated with hypercytokinemia [4].

Genetic changes observed in the H7N9 virus suggest adaptation to mammals, carrying the risk of human-to-human transmission [5]. It has been shown that H7N9 and H7N1 influenza viruses are capable of airborne transmission in a mammalian host (ferret), without losing virulence [6, 7]. These observations suggest the potential for an H7 pandemic in humans, and support pandemic H7 vaccine development. Several H7 inactivated influenza vaccines and live-attenuated influenza vaccines are in clinical development, but have not been highly immunogenic in humans [8–10]. Adjuvanted vaccines have shown improved immunogenicity [11–14]. A recent mix-and-match study demonstrated that a monovalent H7N9 vaccine adjuvanted with AS03 induced a better immune response than the nonadjuvanted or MF59-adjuvanted formulations, when administered to adults according to a 2-dose schedule [14]. Here, we present the findings of a study that evaluated H7N9 vaccine formulations with hemagglutinin (HA) antigen doses of 2.78 and 5.09 µg, given with AS03 adjuvants of different potency and a nonadjuvanted formulation. The doses of AS03-adjuvanted HA antigen were chosen for testing based on a clinical development program by GSK Biologicals with an AS03-adjuvanted split virus H5N1 marketed vaccine.

## METHODS

### Participants, Vaccines, and Study Design

This was a phase I/II, randomized, placebo-controlled, multicenter trial evaluating an H7N9 influenza vaccine (NCT01999842). The trial was approved by independent ethics committees or institutional review boards and was conducted in accordance with the Declaration of Helsinki, the International Conference on Harmonisation Good Clinical Practice guidelines, and regulatory requirements of participating countries. Participants provided written informed consent.

The trial was observer blind and enrolled healthy participants 18–64 years of age in the United States and Canada (inclusion criteria are detailed in Supplementary Text 1). The inactivated, split-virion vaccine, manufactured with a reverse genetic–derived reassortant seed virus developed by World Health Organization Collaborating Centres and References Laboratories from A/Shanghai/2/2013 (H7N9) (GSK Vaccines, Quebec, Canada), was adjuvanted with AS03, an oil-in-water emulsion containing 5.93 mg (AS03_B_) or 11.86 mg (AS03_A_) of DL-α-tocopherol. Participants were randomized 1:1:1:1:1:2 to 1 of 6 groups receiving different HA antigen doses (mixed with adjuvant in groups 1–4) or placebo: (1) 2.78 µg of HA adjuvanted with AS03_B_ (low-dose [LD] HA/AS03_B_), (2) 2.78 µg of HA adjuvanted with AS03_A_ (LD HA/AS03_A_), (3) 5.08 µg of HA adjuvanted with AS03_B_ (medium-dose [MD] HA/AS03_B_), (4) 5.08 µg of HA adjuvanted with AS03_A_ (MD HA/AS03_A_), (5) 10.15 µg HA without AS03 (high-dose [HD] HA nonadjuvanted); or (6) phosphate-buffered saline (placebo).

The antigen doses were less than the initially targeted concentrations of 3.75 and 7.5 µg, because the single radial immunodiffusion assay used to determine the antigen concentration during formulation overestimated the concentration in relation to subsequently available reagents provided by the Center for Biologics Evaluation and Research (CBER) to evaluate vaccine potency. Vaccines were administered twice, 21 days apart, by intramuscular injection in the deltoid muscle.

### Study Objectives

The coprimary immunogenicity objective was to evaluate whether the adjuvanted A/Shanghai/2/2013 (H7N9) vaccines elicited an immune response against the vaccine-homologous virus that met US CBER and European Committee for Medicinal Products for Human Use (CHMP) immunogenicity targets at day 42 (21 days after the second vaccine dose). The primary immunogenicity objective of the study was met if the following criteria were fulfilled for any adjuvanted vaccine formulation: the lower limit of the 98.75% confidence interval was ≥40% for the seroconversion rate (SCR) and ≥70% for seroprotection rate (SPR), and CHMP criteria were met if point estimates were >40% for SCR, >70% for SPR, and >2.5 for the mean geometric increase (MGI). The coprimary safety objective was to describe the safety and reactogenicity of the vaccines up to day 42.

Secondary objectives were: (1) to demonstrate the adjuvant effect for adjuvanted groups that met the primary immunogenicity objective by comparing the immune response of adjuvanted versus nonadjuvanted vaccines measured by hemagglutination inhibition (HI) antibody at day 42 (lower limit of the 98.75% confidence interval for geometric mean titer [GMT] ratio [adjuvanted over nonadjuvanted] >1.5 and SCR difference [adjuvanted minus nonadjuvanted] >10%); (2) to evaluate whether the nonadjuvanted vaccine elicited an immune response against the vaccine-homologous virus that meets CBER and CHMP guidance targets at day 42; (3) to describe the vaccine-homologous and vaccine-heterologous (H7N1 with HA derived from A/mallard/Netherlands/12/2000) HI antibody profiles overall and by age group; (4) to describe the vaccine-homologous (A/Anhui/1/2013 [H7N9] strain) and vaccine-heterologous (H7N1 reverse genetic strain with HA gene derived from A/mallard/Netherlands/12/2000 [H7N3]) microneutralization (MN) antibody profiles in a subset of participants; (5) to describe the safety of the vaccines up to day 385.

### Study End Points and Procedures

Immunogenicity assessments were done with HI and MN assays at baseline (day 0), at 21 days after each dose (days 21 and 42), and at 6 months after the first vaccine dose (day 182), and with HI assay only at 12 months after the first vaccine dose (day 385). HI and MN antibody titers were assessed using horse red blood cells (RBCs). To remove nonspecific agglutinin to horse RBCs and nonspecific virus inhibitors introduced by the hemadsorption step, a receptor-destroying enzyme treatment step was added after horse RBC hemadsorption. Humoral immune response assays were performed by a GSK Biologicals laboratory (HI) and by Viroclinic Biosciences (MN).

The following derived parameters related to the tested vaccine virus were estimated for HI titer: SPR, GMT, SCR, and MGI. SPR was defined as the proportion of participants with reciprocal HI titers ≥40. GMTs were defined as the antilog of the mean of the log_10_-transformed inverse titers. SCR was defined as the proportion of participants with either a prevaccination reciprocal HI titer <10 and a postvaccination reciprocal titer ≥40 or a prevaccination reciprocal HI titer ≥10 and a ≥4-fold increase in postvaccination reciprocal titer. MGI was defined as the geometric mean of the within-participant ratios of the postvaccination to the prevaccination reciprocal HI titer. For MN titer, seropositivity rate and GMT were derived in a similar way as for HI.

Participants recorded solicited injection site and general symptoms between days 0 and 6 after each vaccination in diary cards, collected at the next visit. Symptoms were graded by severity from 1 (mild) to 3 (severe). Grade 3 was defined as “significant pain at rest, preventing everyday activities” for pain, “surface diameter >100 mm” for redness and swelling, temperatures of “≥39.0°C (≥ 102.2°F)” for fever, and “preventing normal activities” for all other solicited symptoms.

Blood samples for safety evaluations were collected on days 0, 7, 21, 28, and 42. Participants recorded unsolicited symptoms (graded by severity) until 21 days after each vaccination. Medically attended adverse events (AEs), potentially immune-mediated disorders (pIMDs), and serious AEs (SAEs) were followed up until the study end. Participants were asked to report immediately any events perceived as serious.

### Statistics

Immunogenicity analyses were performed on the per-protocol cohorts (participants who complied with the protocol, received vaccine, and had assay results available for antibodies against the vaccine-homologous HA antigen at the specified intervals). The safety analysis was descriptive and was performed on the total vaccinated cohort (participants who received ≥1 dose of study vaccine or placebo). Statistical methods are described in detail in Supplementary Text 2.

## RESULTS

### Population Demographics

**Figure 1.**
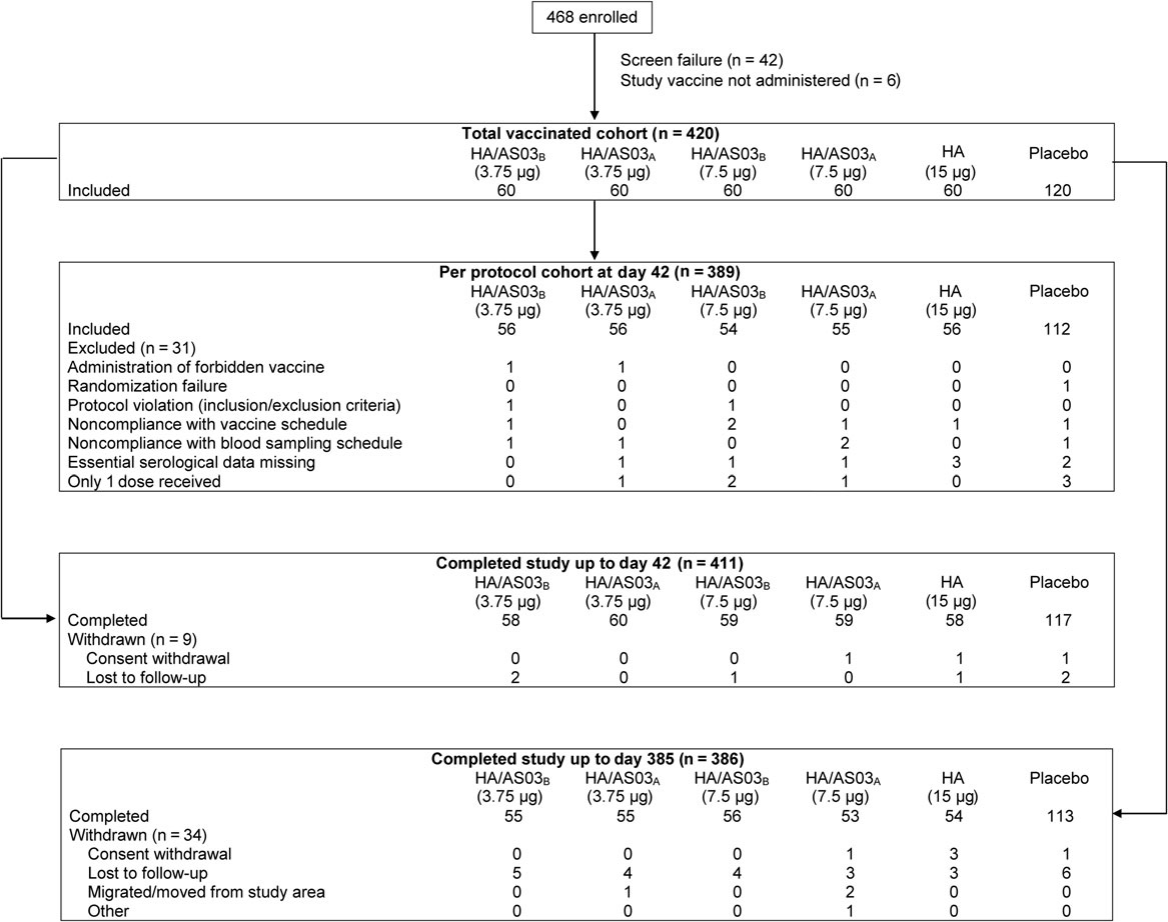
Participant flow chart. Values represent numbers of participants. Abbreviations: HA/AS03_A_, hemagglutinin (HA) adjuvanted with AS03_A_; HA/AS03_B_, hemagglutinin (HA) adjuvanted with AS03_B_.

A total of 420 participants were included in the total vaccinated cohort and 389 in the per-protocol cohort (Figure 1). Demographics were similar in all study groups (Supplementary Table 1). The mean participant age was 40 years, 65% of participants were women, and most (85.5%) were of white European/Caucasian ethnic origin.

### Immunogenicity

CBER and CHMP criteria against the vaccine-homologous virus were met for all adjuvanted vaccines at day 42, 21 days after the second vaccine dose. The SCRs and SPRs were similar in all adjuvanted vaccine groups; SCRs were 85.7%–96.3% and SPRs 91.1%–96.4% (Table 1). The MGI 21 days after the second vaccine dose was highest for the MD HA/AS03_A_ group (25.6), followed by the LD HA/AS03_A_ group (22.8) (Table 1). There was a trend for a higher GMT ratio for the vaccine-homologous virus with the AS03_A_ adjuvant than with the AS03_B_ adjuvant, with a minimal effect of antigen content (Table 2). The GMTs followed the same pattern: 151.1 in the MD HA/AS03_A_ group, 128.0 in the LD HA/AS03_A_ group, 106.2 in the MD HA/AS03_B_ group, and 92.9 in the LD HA/AS03_B_ group (Supplementary Figure S1). Between 12.5% and 19.6% of participants were seropositive before vaccination, although all baseline GMT values were low (only 5 samples had a titer ≥40). After the peak observed on day 42, seropositivity rates and antibody titers declined at day 182 and further at day 385, but GMTs remained above baseline levels in the adjuvanted groups, ranging from 10.8 to 14.3 (Table 1; Supplementary Figure S1). The SPRs at days 182 and 385 were 12.3%–31.5% and 7.8%–19.6%, respectively.

**Table 1.** GMT, SPR, SCR, and MGI for Vaccine-Homologous and Vaccine-Heterologous HI Antibodies (Adapted Per-Protocol Cohort)^a^

	LD HA/AS03_B_		LD HA/AS03_A_		MD HA/AS03_B_		MD HA/AS03_A_		HD HA Nonadjuvanted		Placebo	
	No.	Value (CI)	No.	Value (CI)	No.	Value (CI)	No.	Value (CI)	No.	Value (CI)	No.	Value (CI)
Vaccine Homologous Strain, H7N9^b^												
GMT												
Pre	56	5.9 (5.2–6.8)	56	5.6 (5.1–6.1)	54	5.9 (5.3–6.7)	54	5.9 (5.2–6.7)	56	6.4 (5.5–7.5)	112	5.9 (5.4–6.4)
d 21	56	13.4 (10.7–16.9)	56	15.8 (12.7–19.6)	54	16.5 (12.9–20.9)	55	18.4 (14.2–23.8)	56	10.5 (8.3–13.2)	112	5.6 (5.2–6.0)
d 42	56	92.9 (73.8–116.8)	56	128.0 (96.1–170.7)	54	106.2 (81.5–138.4)	55	151.1 (122.4–186.6)	56	17.2 (13.2–22.5)	112	5.5 (5.2–5.8)
d 182	57	17.4 (14.6–20.8)	55	21.5 (17.4–26.6)	51	20.2 (16.9–24.1)	54	24.3 (20.2–29.3)	54	8.1 (7.0–9.3)	112	5.2 (5.0–5.3)
d 385	51	10.8 (8.9–13.2)	52	12.1 (9.8–15.0)	49	11.7 (9.6–14.2)	51	14.3 (11.6–17.7)	53	6.2 (5.5,6.9)	108	5.1 (5.0–5.2)
SPR, %												
Pre	56	1.8 (.0–9.6)	56	0.0 (.0–6.4)	54	0.0 (.0–6.6)	54	1.9 (.0–9.9)	56	3.6 (.4,12.3)	112	0.9 (.0–4.9)
d 21	56	14.3 (6.4–26.2)	56	16.1 (7.6–28.3)	54	22.2 (12.0–35.6)	55	32.7 (20.7–46.7)	56	10.7 (4.0–21.9)	112	0.0 (.0–3.2)
d 42	56	91.1 (77.2–97.9)	56	91.1 (77.2–97.9)	54	92.6 (78.9–98.6)	55	96.4 (84.6–99.8)	56	28.6 (17.3–42.2)	112	0.0 (.0–3.2)
d 182	57	12.3 (5.1–23.7)	55	23.6 (13.2–37.0)	51	25.5 (14.3–39.6)	54	31.5 (19.5–45.6)	54	0.0 (.0–6.6)	112	0.0 (.0–3.2)
d 385	51	7.8 (2.2–18.9)	52	11.5 (4.4–23.4)	49	10.2 (3.4–22.2)	51	19.6 (9.8–33.1)	53	0.0 (.0–6.7)	108	0.0 (.0–3.4)
SCR, %												
Pre	…	…	…	…	…	…	…	…	…	…	…	…
d 21	56	14.3 (6.4–26.2)	56	14.3 (6.4–26.2)	54	18.5 (9.3–31.4)	54	31.5 (19.5–45.6)	56	7.1 (2.0–17.3)	112	0.0 (.0–3.2)
d 42	56	85.7 (70.4–95.0)	56	89.3 (74.9–97.0)	54	88.9 (74.0–96.9)	54	96.3 (84.4–99.8)	56	23.2 (13.0–36.4)	112	0.0 (.0–3.2)
d 182	57	12.3 (5.1–23.7)	55	23.6 (13.2–37.0)	51	23.5 (12.8–37.5)	53	30.2 (18.3–44.3)	54	0.0 (.0–6.6)	112	0.0 (.0–3.2)
d 385	51	5.9 (1.2–16.2)	52	11.5 (4.4–23.4)	49	8.2 (2.3–19.6)	50	18.0 (8.6–31.4)	53	0.0 (.0–6.7)	108	0.0 (.0–3.4)
MGI												
Pre	…	…	…	…	…	…	…	…	…	…	…	…
d 21	56	2.3 (1.8–2.9)	56	2.8 (2.2–3.5)	54	2.8 (2.2–3.6)	54	3.1 (2.4–4.2)	56	1.6 (1.3–2.0)	112	1.0 (.9–1.0)
d 42	56	15.6 (11.9–20.5)	56	22.8 (16.7–31.0)	54	17.9 (13.2–24.2)	54	25.6 (20.1–32.6)	56	2.7 (2.0–3.5)	112	0.9 (.9–1.0)
d 182	57	2.9 (2.4–3.6)	55	3.7 (3.0–4.7)	51	3.4 (2.7–4.3)	53	4.1 (3.3–5.1)	54	1.3 (1.0–1.5)	112	0.9 (.8–.9)
d 385	51	1.8 (1.5–2.2)	52	2.1 (1.7,2.7)	49	2.0 (1.6–2.5)	50	2.4 (1.9–3.0)	53	0.9 (.8–1.2)	108	0.9 (.8–.9)
Vaccine Heterologous Strain, H7N1^c^												
GMT												
Pre	26	5.5 (4.7–6.3)	26	5.3 (4.9–5.7)	26	5.2 (4.8–5.6)	28	5.3 (4.9–5.8)	27	5.3 (4.7–6.1)	29	5.3 (4.7–6.0)
d 21	26	10.4 (7.5–14.4)	26	11.1 (8.6–14.3)	26	11.1 (8.0–15.5)	28	11.9 (8.4–16.7)	27	6.8 (5.6–8.2)	29	5.2 (4.8–5.8)
d 42	26	59.6 (42.8–83.0)	26	72.8 (48.8–108.5)	26	46.9 (30.2–72.7)	28	66.4 (49.7–88.8)	27	11.9 (7.9–18.1)	29	5.2 (4.8–5.8)
d 182	26	10.7 (8.3–13.7)	26	13.4 (10.1–17.8)	25	11.5 (9.0–14.7)	28	16.0 (12.5–20.3)	26	6.4 (5.2–7.7)	30	5.2 (4.8–5.6)
d 385	23	7.2 (5.6–9.1)	25	8.2 (6.3–10.8)	23	7.3 (5.7–9.3)	27	8.7 (6.8–11.0)	26	5.5 (4.8–6.3)	30	5.0 (5.0–5.0)
SPR, %												
Pre	26	0.0 (.0–13.2)	26	0.0 (.0–13.2)	26	0.0 (.0–13.2)	28	0.0 (.0–12.3)	27	0.0 (.0–12.8)	29	0.0 (.0–11.9)
d 21	26	11.5 (2.4–30.2)	26	3.8 (.1–19.6)	26	11.5 (2.4–30.2)	28	17.9 (6.1–36.9)	27	0.0 (.0–12.8)	29	0.0 (.0–11.9)
d 42	26	73.1 (52.2–88.4)	26	76.9 (56.4–91.0)	26	57.7 (36.9–76.6)	28	75.0 (55.1–89.3)	27	18.5 (6.3–38.1)	29	0.0 (.0–11.9)
d 182	26	7.7 (.9–25.1)	26	11.5 (2.4–30.2)	25	4.0 (.1–20.4)	28	10.7 (2.3–28.2)	26	0.0 (.0–13.2)	30	0.0 (.0–11.6)
d 385	23	0.0 (.0–14.8)	25	4.0 (.1–20.4)	23	4.3 (.1–21.9)	27	0.0 (.0–12.8)	26	0.0 (.0–13.2)	30	0.0 (.0–11.6)
SCR, %												
Pre	…	…	…	…	…	…	…	…	…	…	…	…
d 21	26	11.5 (2.4–30.2)	26	3.8 (.1–19.6)	26	7.7 (.9–25.1)	28	17.9 (6.1–36.9)	27	0.0 (.0–12.8)	29	0.0 (.0–11.9)
d 42	26	69.2 (48.2–85.7)	26	76.9 (56.4–91.0)	26	57.7 (36.9–76.6)	28	75.0 (55.1–89.3)	27	18.5 (6.3–38.1)	29	0.0 (.0–11.9)
d 182	26	7.7 (.9–25.1)	26	11.5 (2.4–30.2)	25	4.0 (.1–20.4)	28	10.7 (2.3–28.2)	26	0.0 (.0–13.2)	30	0.0 (.0–11.6)
d 385	23	0.0 (.0–14.8)	25	4.0 (.1–20.4)	23	4.3 (.1–21.9)	27	0.0 (.0–12.8)	26	0.0 (.0–13.2)	30	0.0 (.0–11.6)
MGI												
Pre	…	…	…	…	…	…	…	…	…	…	…	…
d 21	26	1.9 (1.4–2.7)	26	2.1 (1.6–2.7)	26	2.1 (1.6–2.9)	28	2.2 (1.6–3.2)	27	1.3 (1.0–1.6)	29	1.0 (1.0–1.0)
d 42	26	10.9 (7.4–15.9)	26	13.8 (9.2–20.6)	26	9.0 (5.8–14.0)	28	12.5 (9.3–16.8)	27	2.2 (1.4–3.5)	29	1.0 (1.0–1.0)
d 182	26	1.9 (1.5–2.6)	26	2.5 (1.9–3.4)	25	2.2 (1.7–2.8)	28	3.0 (2.3–3.9)	26	1.2 (.9–1.5)	30	1.0 (.9–1.0)
d 385	23	1.3 (1.0–1.6)	25	1.6 (1.2–2.1)	23	1.4 (1.1–1.8)	27	1.6 (1.3–2.1)	26	1.0 (.8–1.3)	30	0.9 (.8–1.1)

Abbreviations: CI, confidence interval; d, day; GMT, geometric mean titer; HA, hemagglutinin; HD, high-dose; HI, hemagglutination inhibition; LD, low-dose; MD, medium-dose; MGI, mean geometric increase; No., number of participants with available data; Pre, prevaccination; SCR, seroconversion rate; SPR, seroprotection rate.

^a^ The adapted per-protocol cohort includes data for days 0, 21, and 42 from the day 42 per-protocol cohort; day 182 data from the day 182 per-protocol cohort, and day 385 data from the day 385 per-protocol cohort. Parenthetical ranges represent 98.75% CIs for SPR and SCR at day 42 for all adjuvanted groups and 95% CIs for all other values.

^b^ The vaccine homologous strain used for testing was a H7N9 virus developed by reverse genetics with HA and NA genes derived from A/Shanghai/2/2013 (H7N9) and 6 genes from A/PR/8/34.

^c^ The vaccine heterologous strain used for testing was a H7N1 virus developed by reverse genetics with the HA gene derived from A/mallard/Netherlands/12/2000 (H7N3) and 7 genes from A/PR/8/34.

**Table 2.** Adjuvant Effect for Vaccine-Homologous HI Antibodies at 21 Days After the Second Vaccine Dose (Per-Protocol Cohort)

Treatment Group^a^	GMT Ratio (98.75% CI)^b^	SCR Difference (98.75% CI)^c^
LD HA/AS03_B_ (n = 56)	5.72 (3.73–8.77)	62.50 (41.05–77.44)
LD HA/AS03_A_ (n = 56)	7.44 (4.84–11.42)	66.07 (45.30–80.14)
MD HA/AS03_B_ (n = 54)	6.23 (4.05–9.60)	65.67 (44.62–79.90)
MD HA/AS03_A_ (n = 54)	8.84 (5.74–13.61)	73.08 (54.27–85.24)

Abbreviations: CI, confidence interval; GMT, geometric mean titer; HA, hemagglutinin; HD, high-dose; HI, hemagglutination inhibition; LD, low-dose; MD, medium-dose; SCR, seroconversion rate.

^a^ Sample sizes represent number of participants with available data.

^b^ GMT for adjuvanted vaccine (LD or MD HA plus AS03_A_ or AS03_B_)/GMT for nonadjuvanted vaccine (HD HA nonadjuvanted); GMTs were adjusted for baseline value and age.

^c^ SCR for adjuvanted vaccine (LD or MD HA plus AS03_A_ or AS03_B_) minus SCR for nonadjuvanted vaccine (HD HA nonadjuvanted).

**Figure 2.**
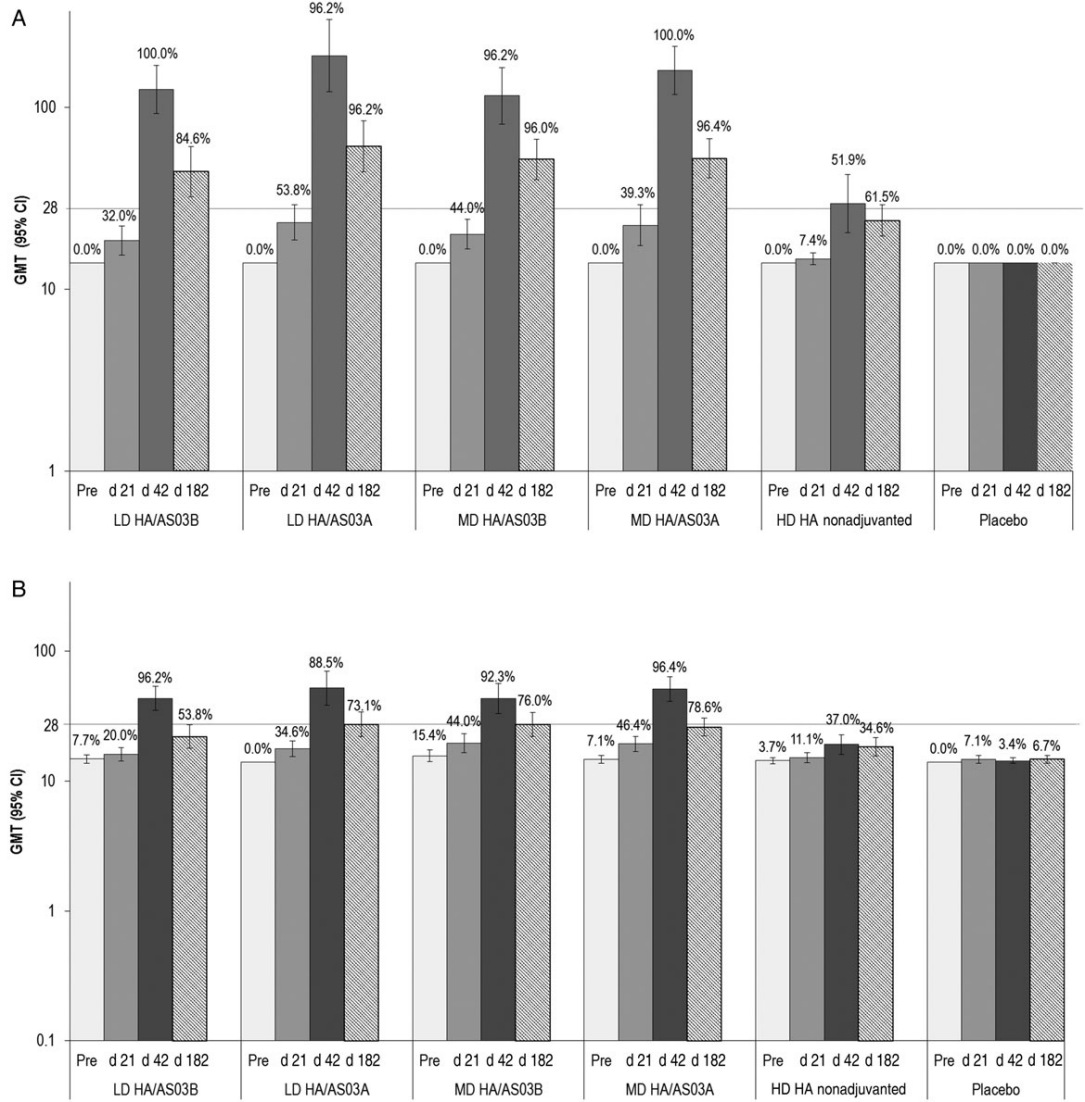
Seropositivity rate and geometric mean titer (GMT) for vaccine-homologous (*A*) and vaccine-heterologous (*B*) microneutralization antibodies (adapted per-protocol cohort). The vaccine homologous strain used for testing was a H7N9 virus developed by reverse genetics with hemagglutinin (HA) and neuraminidase genes derived from A/Anhui/1/2013 (H7N9) and 6 genes from A/PR/8/34. The vaccine heterologous strain was a H7N1 virus developed by reverse genetics with the HA gene derived from A/mallard/Netherlands/12/2000 (H7N3) and 7 genes from A/PR/8/34. Bars represent GMT values; error bars, 95% confidence intervals (CI); percentages above the bars, seropositivity rate; and horizontal gridline, cutoff value for the assay. The adapted per-protocol cohort includes data for days 0, 21, and 42 from the day 42 per-protocol cohort; day 182 data from the day 182 per-protocol cohort; and day 385 data from the day 385 per-protocol cohort. Day 21 was 21 days after the first vaccination, day 42 was 21 days after the second vaccination, and day 182 was 182 days after the first vaccination. Abbreviations: d, day; HD, high-dose; LD, low-dose; MD, medium-dose; Pre, prevaccination measurement.

An immune response against an H7N1 virus was also observed at day 42, albeit at lower levels than against the vaccine-homologous virus. In the adjuvanted groups, 21 days after the second vaccine dose, SCRs were 57.7%–76.9%, MGIs were 9.0–13.8 and GMTs were 46.9–72.8, with the highest response observed in the AS03_A_ adjuvanted groups (Table 1; Figure 2). Immune responses assessed by the MN assay showed a similar kinetic; however, titers remained 1.5–4.3 times higher at day 182 compared with baseline (Figure 2). The vaccine response rate is presented in Supplementary Table S2.

The non-adjuvanted HD HA vaccine elicited a considerably lower immune response against both vaccine-homologous and vaccine-heterologous viruses (Table 1; Figure 2). Adjuvant effect was demonstrated in all adjuvanted groups, as the lower limit of the 98.75% confidence interval for GMT ratio (adjuvanted over nonadjuvanted) exceeded 1.5, and the SCR difference (adjuvanted minus nonadjuvanted) exceeded 10% in all groups at 21 days after the second vaccine dose (Table 2). At days 182 and 385, no study participant had reciprocal HI titers ≥40.

The immune response was low in all vaccine groups after administration of only 1 vaccine dose (Table 1; Figure 2). In a sub-analysis of the homologous immune response by age, the adjuvanted vaccine was immunogenic in both age groups, with SPRs ≥80.0% in participants 41–64 years of age, despite lower GMTs (Table 3). The SPR was 11.5% in participants 41–64 years of age who received the nonadjuvanted HD HA vaccine. The immune response was generally lower in participants who had previously received seasonal influenza vaccine than in nonrecipients. At day 42, GMT values in the adjuvanted vaccine groups were 134.5–220.2 in prior recipients and 70.3–129.0 in nonrecipients. SPR values were 83.9%–96.6% in prior recipients and 95.8%–100% in nonrecipients.

**Table 3.** Vaccine-Homologous HI Antibody Response 21 Days After the Second Vaccine Dose by Age (Per-Protocol Cohort)

Treatment and Age Group^a^	SPR, % (95% CI)	SCR, % (95% CI)	GMT, (95% CI)
LD HA/AS03_B_			
18–40 y (n = 27)	96.3 (81.0–99.9)	96.3 (81.0–99.9)	98.3 (71.8–134.5)
41–64 y (n = 29)	86.2 (68.3–96.1)	75.9 (56.5–89.7)	88.1 (62.0–125.0)
LD HA/AS03_A_			
18–40 y (n = 31)	100 (88.8–100)	100 (88.8–100)	202.4 (150.2–272.6)
41–64 y (n = 25)	80.0 (59.3–93.2)	76.0 (54.9–90.6)	72.6 (46.1–114.3)
MD HA/AS03_B_			
18–40 y (n = 27)	100 (87.2–100)	100 (87.2–100)	156.1 (110.9–219.6)
41–64 y (n = 27)	85.2 (66.3–95.8)	77.8 (57.7–91.4)	72.2 (50.0–104.4)
MD HA/AS03_A_			
18–40 y (n = 31)^b^	100 (88.8–100)	100 (88.4–100)	173.0 (137.2–218.0)
41–64 y (n = 24)	91.7 (73.0–99.0)	91.7 (73.0–99.0)	126.9 (85.9–187.6)
HD HA nonadjuvanted			
18–40 y (n = 30)	43.3 (25.5–62.6)	36.7 (19.9–56.1)	21.9 (14.9–32.2)
41–64 y (n = 26)	11.5 (2.4–30.2)	7.7 (.9–25.1)	13.0 (9.0–18.9)
Placebo			
18–40 y (n = 61)	0.0 (.0–5.9)	0.0 (.0–5.9)	5.1 (4.9–5.3)
41–64 y (n = 51)	0.0 (.0–7.0)	0.0 (.0–7.0)	5.9 (5.3–6.6)

Abbreviations: CI, confidence interval; GMT, geometric mean titer; HA, hemagglutinin; HD, high-dose; HI, hemagglutination inhibition; LD, low-dose; MD, medium-dose; SCR, seroconversion rate; SPR, seroprotection rate.

^a^ Sample sizes represent number of participants with available data (for SCR calculation, number with available data both before and after vaccination).

^b^ For SCR, n = 30.

### Safety and Reactogenicity

Pain was the most common injection site solicited symptom, occurring more frequently in the adjuvanted vaccine groups than in the HD HA nonadjuvanted group (Table 4). Redness and swelling at the injection site occurred in 3.3%–6.7% of participants receiving vaccines adjuvanted with AS03_B_ and in 13.3%–18.3% of those receiving vaccines adjuvanted with AS03_A_ (Table 4). Grade 3 injection site solicited symptoms were reported by up to 6.7% of the participants in the adjuvanted groups and by none of the participants in the nonadjuvanted and placebo groups. Fatigue, headache, and muscle ache were the most frequently reported solicited general symptoms (Table 4). Fatigue and muscle ache occurred in 45.0%–55.0% of participants in all adjuvanted groups, compared with 25.4%–28.8% in the HD HA nonadjuvanted and 25.0%–20.8% in the placebo group. Fever occurred infrequently and at a similar rate across all study groups.

**Table 4.** Safety Outcomes Reported (Total Vaccinated Cohort)

Outcome	Participants With Outcome, %^a^					
	LD HA/AS03_B_ (n = 60)	LD HA/AS03_A_ (n = 60)	MD HA/AS03_B_ (n = 60)	MD HA/AS03_A_ (n = 60)	HD HA Nonadjuvanted (n = 60)^b^	Placebo (n = 120)
Solicited symptoms during 7-d postvaccination period^c^						
Injection site symptoms						
Pain	88.3 (77.4–95.2)	91.7 (81.6–97.2)	90.0 (79.5–96.2)	98.3 (91.1–100)	40.7 (28.1–54.3)	20.0 (13.3–28.3)
Redness	3.3 (.4–11.5)	13.3 (5.9–24.6)	3.3 (.4–11.5)	13.3 (5.9–24.6)	1.7 (.0–9.1)	0.0 (.0–3.0)
Swelling	3.3 (.4–11.5)	18.3 (9.5–30.4)	6.7 (1.8–16.2)	15.0 (7.1–26.6)	0.0 (.0–6.1)	0.0 (.0–3.0)
General symptoms						
Fatigue	50.0 (36.8–63.2)	48.3 (35.2–61.6)	50.0 (36.8–63.2)	53.3 (40.0–66.3)	25.4 (15.0–38.4)	25.0 (17.5–33.7)
Fever	3.3 (.4–11.5)	5.0 (1.0–13.9)	1.7 (.0–8.9)	6.7 (1.8–16.2)	1.7 (.0–9.1)	4.2 (1.4–9.5)
Gastrointestinal symptoms	31.7 (20.3–45.0)	23.3 (13.4–36.0)	20.0 (10.8–32.3)	15.0 (7.1–26.6)	18.6 (9.7–30.9)	19.2 (12.6–27.4)
Headache	51.7 (38.4–64.8)	56.7 (43.2–69.4)	45.0 (32.1–58.4)	48.3 (35.2–61.6)	30.5 (19.2–43.9)	37.5 (28.8–46.8)
Joint pain	26.7 (16.1–39.7)	30.0 (18.8–43.2)	20.0 (10.8–32.3)	30.0 (18.8–43.2)	13.6 (6.0–25.0)	9.2 (4.7–15.8)
Muscle ache	48.3 (35.2–61.6)	53.3 (40.0–66.3)	45.0 (32.1–58.4)	55.0 (41.6–67.9)	28.8 (17.8–42.1)	20.8 (14.0–29.2)
Shivering	18.3 (9.5–30.4)	33.3 (21.7–46.7)	15.0 (7.1–26.6)	30.0 (18.8–43.2)	13.6 (6.0–25.0)	10.0 (5.3–16.8)
Sweating	15.0 (7.1–26.6)	21.7 (12.1–34.2)	13.3 (5.9–24.6)	18.3 (9.5–30.4)	8.5 (2.8–18.7)	8.3 (4.1–14.8)
Unsolicited symptoms during 21-d postvaccination period^d^						
All	38.3 (26.1–51.8)	48.3 (35.2–61.6)	48.3 (35.2–61.6)	48.3 (35.2–61.6)	48.3 (35.2–61.6)	39.2 (30.4–48.5)
Related to vaccine	6.7 (1.8–16.2)	11.7 (4.8–22.6)	13.3 (5.9–24.6)	13.3 (5.9–24.6)	6.7 (1.8–16.2)	1.7 (.2–5.9)
Medically attended AEs up to 385 d after first vaccine dose^e^						
All	38.3 (26.1–51.8)	41.7 (29.1–55.1)	45.0 (32.1–58.4)	36.7 (24.6–50.1)	45.0 (32.1–58.4)	38.3 (29.6–47.6)
SAEs up to 385 d after first vaccine dose						
All	1.7 (.0–8.9)	0.0 (.0–6.0)	5.0 (1.0–13.9)	6.7 (1.8–16.2)	3.3 (.4–11.5)	2.5 (.5–7.1)

Abbreviations: AEs, adverse events; HA, hemagglutinin; HD, high-dose; LD, low-dose; MD, medium-dose; SAEs, serious AEs.

^a^ Data represent percentage of study participants reporting the event at least once; sample sizes, number of study participants with ≥1 documented dose.

^b^ Note: n = 59 for solicited symptoms.

^c^ All solicited injection site symptoms were considered related to vaccination.

^d^ Unsolicited symptoms were spontaneously reported.

^e^ Including hospitalizations and visits to emergency rooms or medical practitioners.

Most solicited AEs resolved spontaneously. Grade 3 solicited and unsolicited events occurred at a low rate and few unsolicited AEs were considered related to vaccination (Table 4). Twenty SAEs were reported in 13 participants up to the study end, and none of them were assessed as vaccination related. Nine pregnancies occurred during the study. One participant (exposed to the vaccine during the first trimester) underwent elective abortion, not related to the vaccination. For the other 8 pregnancies, the exposure to study vaccine occurred before the pregnancy; 6 gave birth to live neonates, and for 2 the outcome was unknown. One potentially immune-mediated disorder assessed by the investigator as not related to vaccination—autoimmune thyroiditis—was reported 303 days after administration of the second dose of placebo. No deaths were reported during the study. For hematological and biochemical parameters, results outside of the normal laboratory range were evenly distributed across all time points (including baseline) and vaccine groups, and no clear clinical trends were observed (Supplementary Tables S3 and S4).

## DISCUSSION

The results of this phase I/II randomized, placebo-controlled trial showed that 2 doses of the H7N9 AS03-adjuvanted vaccine elicited a robust immune response in healthy adults up to 64 years of age, with an acceptable safety profile. Adjuvantation with AS03 enabled an immune response that satisfied regulatory acceptance criteria at antigen-sparing concentrations of HA, a prerequisite for a pandemic influenza vaccine. A dose as low as 2.8 µg HA elicited a robust HI antibody response.

HI responses were low after the first vaccine dose in all vaccine groups, indicating that 2 doses are required to induce an adequate immune response. Three weeks after the second vaccine dose, SPRs and SCRs against the vaccine-homologous virus were ≥85.7% in the adjuvanted vaccine groups. The non-adjuvanted vaccine elicited a poor immune response, and an adjuvant effect was demonstrated in all adjuvanted groups in terms of GMT ratio and SCR difference. The GMT and MGI for the AS03-adjuvanted vaccines against the vaccine-homologous virus were highest in the MD HA/AS03_A_ group, followed by the LD HA/AS03_A_ group, the HD HA/AS03_B_ group, and finally the LD HA/AS03_B_ group. Thus, the potency of the AS03 adjuvant (11.86 or 5.93 mg tocopherol in AS03_A_ or AS03_B_, respectively) seems to have more influence than antigen content on immunogenicity. This was also observed in a study of a H7N9 HA antigen produced by a different manufacturer mixed with GSK's AS03_A_ at the point of use [14] and in a study of with the AS03-adjuvanted H5N1 pandemic vaccine [15]. Immune responses in older participants (aged 41–64 years) were generally lower than in younger participants (aged 18–40 years) and lower in participants who had previously received seasonal influenza vaccine than in nonrecipients, consistent with findings in other studies of pandemic influenza vaccines [13, 14, 16, 17].

Up to 19.6% of study participants were seropositive before vaccination. Detectable levels of HI antibody in the general population before vaccination with pandemic influenza vaccines have been reported in several studies [18–20], suggesting either natural immune response to previous exposure or cross-reactivity between virus strains. Previous exposure is unlikely, because the H7N9 virus circulated in China and no infections in humans were reported in the North American population [21]. In addition, human antibody response to this strain has been shown to be very poor [22]. The same study suggests cross-reactivity of H1 and H3 seasonal influenza subtypes to the H7N9 virus, and this is the most likely explanation of our finding. The CD8^+^ T cells to seasonal influenza are reported to recognize H7N9 epitopes and to exhibit cross-reactivity with the H7N9 virus [23]. In our study, cross-reactivity could originate either from previous infection or seasonal vaccination. Of note, a total of 218 study participants had received vaccination against influenza within the previous 3 seasons, although the virus strain to which the participants were previously exposed was not documented.

The study shows that the AS03 adjuvant enhances the immune response against H7 antigens at antigen-sparing doses. Previous studies of H7 vaccines that were nonadjuvanted or adjuvanted with aluminum hydroxide have shown poor immunogenicity in humans [8, 9]. Clinical studies of adjuvanted H7N9 vaccines from different manufacturers have found higher immunogenicity compared to nonadjuvanted formulations [11–14]. A phase II study of an H7N9 vaccine mixed at the point of use with MF59 adjuvant resulted in seroconversion in 59% of participants with the LD HA dose; higher antigen doses did not elicit an increase in immunogenicity [13]. A recent phase II study compared different doses of an H7N9 vaccine mixed at the point of use with AS03_A_ or MF59 or administered without an adjuvant [14]. As in the present study, the immune response with all formulations was low after 1 vaccine dose. After 2 doses, the immune response was superior with the AS03-adjuvanted formulations compared with the MF59-adjuvanted or standard or high-dose nonadjuvanted formulations [14]. For AS03-adjuvanted vaccines, similar immunogenicity was attained with antigen content varying between 3.75, 7, and 15 µg of HA [14].

The present study also demonstrated cross-reactivity of the vaccine against a vaccine-heterologous strain. Phylogenetic analysis has shown a high degree of homology in the HA gene sequence of various H7 viruses [24]. An AS03-adjuvanted H7N1 vaccine, engineered by reverse genetics from an H7N3 virus, has been developed by GSK Vaccines, and therefore the present study evaluated cross-reactivity against an H7N1 vaccine-heterologous virus. Robust cross-reactivity was seen, with SPRs ranging from 57.7% to 76.9%. In preclinical studies in mice, an H7N1/AS03 vaccine elicited antibodies cross-reacting with H7N9, H7N7, and H7N3 viruses [25], and an H7N9 virus-like particle vaccine produced in insect cells using a baculovirus vector elicited antibodies cross-reacting with an H7N3 virus [26].

To our knowledge, this is the first report on the immune response against H7N9 in humans beyond day 42 after vaccination. Based on the blood sampling schedule, HI antibody titers against the vaccine-homologous virus peaked at 21 days after the second dose. At day 182, a notable decrease in GMTs was observed, although of different magnitude across groups. However, seropositive rates remained above 90% at day 182 and above 70% at day 385 in all adjuvanted groups, compared with 50.0% and 22.6% in the nonadjuvanted group and 4.5% and 1.9% in the placebo group. In addition, recent studies seem to suggest that a robust immune response to the HA head and stalk domains, as measured with enzyme-linked immunosorbent assay, may be induced even in the absence of HI and MN response [27], so the clinical relevance of the decline in antibody titers is not clear. The observed kinetics of the immune response in adjuvanted groups in our study is similar to that elicited by other influenza vaccines, in particular the adjuvanted H5N1 vaccines [28], for which a strong anamnestic response was elicited by a heterologous booster dose, up to 3 years after priming [29, 30].

The H7N9 AS03-adjuvanted vaccine was generally well tolerated. Pain was the most common solicited injection site symptom, as observed in other studies of adjuvanted influenza vaccines [12–17, 19, 31, 32]. Tolerability seemed to be acceptable, as most participants returned for the second vaccine dose. Most AEs were low grade and resolved spontaneously. None of the SAEs was assessed as related to vaccination.

In this study, a trend was observed for higher reactogenicity with AS03_A_-adjuvanted vaccines than with AS03_B_ formulations. However, all formulations were well tolerated and no safety signal was identified during the study. The use of the AS03_A_ adjuvant led to an improved immune response compared with that induced by AS03_B_-adjuvanted formulations, so the risk-benefit ratio in using a high potency adjuvant seems clinically acceptable.

Pandemic preparedness strategies include development of vaccines that are antigen sparing, because the time available to produce sufficient antigen to provide effective vaccine coverage is likely to be limited. In addition, vaccines that elicit a broad cross-reactive immune response are needed to allow pre-pandemic priming and use of prime-boost vaccination schedules. Formulation of pandemic vaccines with an effective adjuvant is an accepted strategy to accomplish both goals [33] and to ensure adequate immunogenicity for the elderly and those with reduced immune responsiveness due to concurrent illness.

The assessment of immunogenicity for this study is based on HI and MN assays; no additional evaluation such as cell-mediated immunity was performed which could bring additional information but for which no regulatory acceptance criteria have been established. No formal comparisons between adjuvanted vaccine groups were performed, and analyses were descriptive. These could constitute potential limitations to the study. Nevertheless, the type I error was adjusted for the evaluation of the primary immunogenicity objective, as well as the secondary objective related to adjuvant effect.

In conclusion, 2 doses of an AS03-adjuvanted H7N9 vaccine induce a robust anti-H7 immune response with an acceptable safety profile. When balancing antigen sparing with effective immunization, a 2.78-µg antigen dose (or 3.75 µg to align with the already licensed H5N1 vaccines made by the same process) with AS03_A_ adjuvantation seems the most desirable formulation. This vaccine candidate could be beneficially deployed should the H7N9 virus acquire the ability for sustained human-to-human transmission or to protect persons at risk.

## Supplementary Material

Supplementary Data
