# Supplementary material for: Immunogenicity and Safety of an AS03-Adjuvanted H7N9 Pandemic Influenza Vaccine in a Randomized Trial in Healthy Adults
Source: J Infect Dis. 2016 Sep 7;214(11):1717–27. doi: 10.1093/infdis/jiw414 (PMC5144728; doi:10.1093/infdis/jiw414)
Supplement: Supplementary Data [file jiw414_Supplementary_Data.zip › jiw414supp.doc]

**SUPPLEMENTARY MATERIAL**

## Supplementary text 1. Selection of study population

*Inclusion criteria for enrolment*

• Male or female adults who are 18 to 64 years of age (inclusive) at the time of first study vaccination.

• Written informed consent obtained from subject.

• Subjects who the investigator believes can and will comply with the requirements of the protocol.

• Healthy subjects as established by medical history and physical examination.

• Access to a consistent means of telephone contact.

• For subjects who undergo a screening visit: results of all safety laboratory tests obtained at the screening visit must be within reference ranges. Results of any repeat testing cannot be used to qualify a subject for enrolment.

• Female subjects of non-childbearing potential may be enrolled in the study. Non-childbearing potential is defined as current tubal ligation, hysterectomy, ovariectomy or post-menopause.

• Female subjects of childbearing potential may be enrolled in the study, if they

-have practiced adequate contraception for 30 days prior to vaccination, and

- have a negative pregnancy test on the day of vaccination, and

- agree to continue to practice adequate contraception until 2 months after the last dose administered.

*Exclusion criteria for enrolment*

• Presence or evidence of neurological or psychiatric diagnoses which, although stable, are deemed by the investigator to render the potential subject unable/unlikely to provide accurate safety reports.

• Presence or evidence of substance abuse.

• Diagnosed with cancer, or treatment for cancer within three years.

• Diagnosed with excessive daytime sleepiness, or narcolepsy; or history of narcolepsy in a subject’s parent, sibling or child.

• Presence of a temperature ≥ 38.0ºC (≥100.4ºF), or acute symptoms greater than “mild” severity on the scheduled date of first vaccination.

• Any confirmed or suspected immunosuppressive or immunodeficient condition including history of human immunodeficiency virus (HIV) infection.

• Receipt of systemic glucocorticoids within 30 days prior to the first dose of study vaccine/placebo, or any other cytotoxic, immunosuppressive or immune-modifying drugs within 6 months of first study vaccine/ placebo dose.

• Any significant disorder of coagulation or treatment with warfarin derivatives or heparin.

• An acute evolving neurological disorder or Guillain Barré Syndrome within 42 days of receipt of prior seasonal or pandemic influenza vaccine.

• Administration of an inactive vaccine within 14 days or of a live attenuated vaccine within 30 days before the first dose of study vaccine/placebo.

• Planned administration of any vaccine other than the study vaccine/placebo before blood sampling at the Day 42 visit.

• Previous administration of any H7 vaccine or physician-confirmed H7 disease.

• Use of any investigational or non-registered product other than the study vaccine within 30 days preceding the first dose of study vaccine/placebo, or planned use during the study period.

• Receipt of any immunoglobulins and/or any blood products within 90 days before the first dose of study vaccine/placebo, or planned administration of any of these products during the study period.

• Any known or suspected allergy to any constituent of influenza vaccines or component used in the manufacturing process of the study vaccine including a history of anaphylactic-type reaction to consumption of eggs; or a history of severe adverse reaction to a previous influenza vaccine.

• Known pregnancy or a positive urine beta-human chorionic gonadotropin (β-hCG) test result before the first dose of study vaccine/placebo.

• Lactating or nursing women.

• Any condition which, in the opinion of the investigator, prevents the subject from participating in the study.

A complete list of the study locations and the protocol summary are available at <https://clinicaltrials.gov/ct2/show/NCT01999842> or <http://www.gsk-clinicalstudyregister.com/study/201072> .

**Supplementary text 2. Statistical methods**

Assuming enrolment of 60 participants per group, a non-evaluable rate of 5%, and assuming the observed SCR and SPR were 90%, the study had 88.80% power to meet CBER criteria (LL of the 98.75% CI of SPR ≥70% and a SCR of >40% in any adjuvanted vaccine group, calculated using PASS 2005, one-sided type I error of 0.625%). Under the same assumptions, the study had >99.99% power to meet CHMP criteria (a SPR ≥70%, a SCR of >40% and MGI>2.5 in any adjuvanted vaccine group, calculated using PASS 2005, one-sided type I error of 50%). Evaluation of the adjuvant effect was planned for the groups meeting CBER and CHMP criteria. If the primary objective was met in all adjuvanted vaccine groups, the power for adjuvant effect evaluation was 95.03% for each adjuvanted group evaluation (95.88% for GMT ratio evaluation assuming a GMT ratio of 5 under an alternative hypothesis and an alpha of 0.625% and 99.15% for SCR difference evaluation assuming a SCR difference of 50% under an alternative hypothesis and an alpha of 0.625%). The type I error was split equally for each adjuvanted group evaluation for the primary immunogenicity objective, as well as the adjuvant effect evaluation, so that the overall type I error was controlled at a level of 5%. All other analyses were descriptive.

Vaccine-heterologous antibodies were evaluated in a subset of approximately 180 participants (30 per group). A sub-randomization generated subsets of ~50% of participants in each vaccine group and ~25% of participants from the placebo group, accounting for age group (18–40 and 41–64 years).

The technical cut-off value for HI and MN titers was set at 1:10 and 1:28, respectively. A seropositive participant was defined as having a titer greater than or equal to the cut-off value; a seronegative participant was given an arbitrary value of half the cut-off for the GMT calculation.

For the primary immunogenicity endpoint at Day 42, point estimates with two-sided CIs were calculated (exact 98.75% for SCR and SPR, and 95% for MGI). To demonstrate the adjuvant effect (secondary endpoint), the GMT ratio and its two-sided 98.75% CI for the adjuvanted over non-adjuvanted vaccine were computed after fitting an ANCOVA model on the log_10_ transformation of the titers, including the vaccine group as fixed effect and pre-vaccination HI titers and participant age as covariates. The asymptotic standardized 98.75% CI for the difference in SCR between adjuvanted and non-adjuvanted vaccine was also computed. For the other secondary objectives, 95% CIs were calculated. As the SCR, VRR and MGI calculations required baseline results, the analysis of these endpoints excluded participants with missing or non-evaluable measurements at baseline.

**Supplementary Table S1.** Participant demographics (total vaccinated cohort)

|  | **LD HA/AS03_B_** | **LD HA/AS03_A_** | **MD HA/AS03_B_** | **MD HA/AS03_A_** | **HD Ha unadj** | **Placebo** | **Total** |
| --- | --- | --- | --- | --- | --- | --- | --- |
| N | 60 | 60 | 60 | 60 | 60 | 120 | 420 |
| Mean age (SD), years | 41.4 (12.6) | 39.8 (14.1) | 39.8 (13.2) | 40.3 (13.1) | 39.6 (13.9) | 39.9 (13.5) | 40.1 (13.3) |
| Female gender, n (%) | 40 (66.7) | 39 (65.0) | 41 (68.3) | 39 (65.0) | 34 (56.7) | 80 (66.7) | 273 (65.0) |
| Geographic ancestry, n (%) | | | | | | | |
| African/African American | 5 (8.3) | 6 (10.0) | 6 (10.0) | 6 (10.0) | 6 (10.0) | 11 (9.2) | 40 (9.5) |
| American Indian or Alaskan Native | 1 (1.7) | 0 | 0 | 0 | 1 (1.7) | 2 (1.7) | 4 (1.0) |
| Asian | 2 (3.3) | 1 (1.7) | 0 | 0 | 1 (1.7) | 1 (0.8) | 5 (1.2) |
| Native Hawaiian or other Pacific Islander | 0 | 0 | 0 | 0 | 0 | 0 | 0 |
| White (Arabic or North African) | 0 | 0 | 0 | 0 | 1 (1.7) | 1 (0.8) | 2 (0.5) |
| White (Caucasian or European) | 52 (86.7) | 52 (86.7) | 51 (85.0) | 53 (88.3) | 50 (83.3) | 101 (84.2) | 359 (85.5) |
| Other | 0 | 1 (1.7) | 3 (5.0) | 1 (1.7) | 1 (1.7) | 4 (3.3) | 10 (2.4) |

HD: high dose; LD: low dose; MD: medium dose; N: number of participants in each group; n (%): number (percentage) of individuals in each category.

**Supplementary table S2.** Vaccine response rate (VRR) at Day 0, Day 21, Day 42 and Day 182 assessed by MN assay (adapted per protocol cohort)

|  |  | LD HA/AS03_B_ | | LD HA/AS03_A_ | | MD HA/AS03_B_ | | MD HA/AS03_A_ | | HD HA unadj | | Placebo | |
| --- | --- | --- | --- | --- | --- | --- | --- | --- | --- | --- | --- | --- | --- |
|  |  | N | % (95% CI) | N | % (95% CI) | N | % (95% CI) | N | % (95% CI) | N | % (95% CI) | N | % (95% CI) |
| Anhui/1/2013  (vaccine homologous, H7N9) | Pre-vac. | - |  | - |  | - |  | - |  | - |  | - |  |
|  | Day 21 | 25 | 8.0 (1.0; 26.0) | 26 | 19.2 (6.6; 39.4) | 25 | 8.0 (1.0; 26.0) | 28 | 25.0 (10.7; 44.9) | 27 | 0.0 (0.0; 12.8) | 28 | 0.0 (0.0; 12.3) |
|  | Day 42 | 26 | 92.3 (74.9; 99.1) | 26 | 92.3 (74.9; 99.1) | 26 | 88.5 (69.8; 97.6) | 28 | 92.9 (76.5; 99.1) | 27 | 33.3 (16.5; 54.0) | 29 | 0.0 (0.0; 11.9) |
|  | Day 182 | 26 | 53.8 (33.4; 73.4) | 26 | 69.2 (48.2; 85.7) | 25 | 64.0 (42.5; 82.0) | 28 | 64.3 (44.1; 81.4) | 26 | 15.4 (4.4; 34.9) | 30 | 0.0 (0.0; 11.6) |
| A/mallard/Netherlands/12/2000  (vaccine heterologous, H7N1) | Pre-vac. | - |  | - |  | - |  | - |  | - |  | - |  |
|  | Day 21 | 25 | 0.0 (0.0; 13.7) | 26 | 0.0 (0.0; 13.2) | 25 | 4.0 (0.1; 20.4) | 28 | 0.0 (0.0; 12.3) | 27 | 0.0 (0.0; 12.8) | 28 | 0.0 (0.0; 12.3) |
|  | Day 42 | 26 | 53.8 (33.4; 73.4) | 26 | 61.5 (40.6; 79.8) | 26 | 46.2 (26.6; 66.6) | 28 | 67.9 (47.6; 84.1) | 27 | 7.4 (0.9; 24.3) | 29 | 0.0 (0.0; 11.9) |
|  | Day 182 | 26 | 7.7 (0.9; 25.1) | 26 | 19.2 (6.6; 39.4) | 25 | 12.0 (2.5; 31.2) | 28 | 10.7 (2.3; 28.2) | 26 | 3.8 (0.1; 19.6) | 30 | 0.0 (0.0; 11.6) |

VRR was defined as the proportion of participants with either a pre-vaccination reciprocal MN titer <28 and a post-vaccination reciprocal titer ≥56, or a pre-vaccination reciprocal MN titer ≥28 and at least a 4-fold increase in post-vaccination reciprocal titer against the tested virus.

CI: confidence interval; HD: high dose; LD: low dose; MD: medium dose; MN: microneutralisation; N: Number of participants with both pre and post results available; % percentage of responders

The adapted per protocol cohort includes Day 0, Day 21 and Day 42 data from the Day 42 per protocol cohort; Day 182 data from the Day 182 per protocol cohort.

**Supplementary table S3.** Distribution of biochemistry with respect to normal laboratory ranges (total vaccinated cohort)

|  |  |  | LD HA/AS03B | | | |  |  | LD HA/AS03A | | |  | MD HA/AS03B | | | |  | MD HA/AS03A | | | |  | | HD Ha unadj | | | |  | Placebo | | | |
| --- | --- | --- | --- | --- | --- | --- | --- | --- | --- | --- | --- | --- | --- | --- | --- | --- | --- | --- | --- | --- | --- | --- | --- | --- | --- | --- | --- | --- | --- | --- | --- | --- |
|  | |  |  | Below | Within | Above |  |  | Below | Within | Above |  |  | Below | Within | Above |  |  | Below | Within | Above | |  |  | Below | Within | Above |  |  | Below | Within | Above |
| Laboratory parameter | Timing |  | N | % | % | % |  | N | % | % | % |  | N | % | % | % |  | N | % | % | % | |  | N | % | % | % |  | N | % | % | % |
| Alanine Amino-transferase | Pre-vac. |  | 60 | 0.0 | 100.0 | 0.0 |  | 60 | 0.0 | 88.3 | 10.0 |  | 60 | 0.0 | 100.0 | 0.0 |  | 60 | 0.0 | 95.0 | 5.0 | |  | 60 | 0.0 | 96.7 | 3.3 |  | 120 | 0.0 | 95.8 | 4.2 |
|  | Day 7 |  | 59 | 0.0 | 100.0 | 0.0 |  | 60 | 0.0 | 95.0 | 5.0 |  | 60 | 0.0 | 96.7 | 1.7 |  | 59 | 0.0 | 96.6 | 3.4 | |  | 59 | 0.0 | 98.3 | 1.7 |  | 118 | 0.0 | 94.9 | 4.2 |
|  | Day 21 |  | 60 | 0.0 | 93.3 | 6.7 |  | 60 | 0.0 | 91.7 | 8.3 |  | 60 | 0.0 | 96.7 | 3.3 |  | 60 | 0.0 | 93.3 | 5.0 | |  | 58 | 0.0 | 100.0 | 0.0 |  | 117 | 0.0 | 93.2 | 6.0 |
|  | Day 28 |  | 60 | 0.0 | 96.7 | 3.3 |  | 59 | 0.0 | 91.5 | 8.5 |  | 60 | 0.0 | 100.0 | 0.0 |  | 59 | 0.0 | 98.3 | 1.7 | |  | 58 | 0.0 | 96.6 | 3.4 |  | 117 | 0.0 | 92.3 | 7.7 |
|  | Day 42 |  | 58 | 0.0 | 96.6 | 3.4 |  | 59 | 0.0 | 94.9 | 3.4 |  | 59 | 0.0 | 98.3 | 1.7 |  | 59 | 0.0 | 98.3 | 1.7 | |  | 58 | 0.0 | 96.6 | 1.7 |  | 117 | 0.0 | 94.0 | 6.0 |
| Aspartate Amino-transferase | Pre-vac. |  | 60 | 0.0 | 100.0 | 0.0 |  | 60 | 0.0 | 96.7 | 1.7 |  | 60 | 0.0 | 96.7 | 3.3 |  | 60 | 0.0 | 95.0 | 5.0 | |  | 60 | 0.0 | 98.3 | 1.7 |  | 120 | 0.0 | 95.8 | 4.2 |
|  | Day 7 |  | 59 | 0.0 | 100.0 | 0.0 |  | 60 | 0.0 | 98.3 | 1.7 |  | 60 | 0.0 | 95.0 | 3.3 |  | 59 | 0.0 | 96.6 | 3.4 | |  | 59 | 0.0 | 100.0 | 0.0 |  | 118 | 0.0 | 95.8 | 3.4 |
|  | Day 21 |  | 60 | 0.0 | 96.7 | 3.3 |  | 60 | 0.0 | 98.3 | 1.7 |  | 60 | 0.0 | 98.3 | 1.7 |  | 60 | 0.0 | 95.0 | 3.3 | |  | 58 | 0.0 | 100.0 | 0.0 |  | 117 | 0.0 | 95.7 | 3.4 |
|  | Day 28 |  | 60 | 0.0 | 96.7 | 3.3 |  | 59 | 0.0 | 96.6 | 3.4 |  | 60 | 0.0 | 96.7 | 1.7 |  | 59 | 0.0 | 98.3 | 1.7 | |  | 58 | 0.0 | 98.3 | 1.7 |  | 117 | 0.0 | 94.9 | 5.1 |
|  | Day 42 |  | 58 | 0.0 | 96.6 | 3.4 |  | 59 | 0.0 | 96.6 | 1.7 |  | 59 | 0.0 | 100.0 | 0.0 |  | 59 | 0.0 | 98.3 | 1.7 | |  | 58 | 0.0 | 98.3 | 0.0 |  | 117 | 0.0 | 96.6 | 3.4 |
| Creatinine | Pre-vac. |  | 60 | 3.3 | 96.7 | 0.0 |  | 60 | 5.0 | 93.3 | 0.0 |  | 60 | 8.3 | 91.7 | 0.0 |  | 60 | 15.0 | 81.7 | 3.3 | |  | 60 | 6.7 | 91.7 | 1.7 |  | 120 | 10.8 | 89.2 | 0.0 |
|  | Day 7 |  | 59 | 5.1 | 91.5 | 3.4 |  | 60 | 8.3 | 90.0 | 1.7 |  | 60 | 6.7 | 91.7 | 0.0 |  | 59 | 13.6 | 84.7 | 1.7 | |  | 59 | 13.6 | 86.4 | 0.0 |  | 118 | 7.6 | 91.5 | 0.0 |
|  | Day 21 |  | 60 | 5.0 | 93.3 | 1.7 |  | 60 | 6.7 | 93.3 | 0.0 |  | 60 | 6.7 | 93.3 | 0.0 |  | 60 | 13.3 | 85.0 | 0.0 | |  | 58 | 8.6 | 89.7 | 1.7 |  | 117 | 10.3 | 88.0 | 0.9 |
|  | Day 28 |  | 60 | 3.3 | 93.3 | 3.3 |  | 59 | 5.1 | 93.2 | 1.7 |  | 60 | 8.3 | 91.7 | 0.0 |  | 59 | 6.8 | 93.2 | 0.0 | |  | 58 | 5.2 | 93.1 | 1.7 |  | 117 | 6.0 | 92.3 | 1.7 |
|  | Day 42 |  | 58 | 6.9 | 91.4 | 1.7 |  | 59 | 6.8 | 89.8 | 1.7 |  | 59 | 6.8 | 93.2 | 0.0 |  | 59 | 10.2 | 89.8 | 0.0 | |  | 58 | 10.3 | 86.2 | 1.7 |  | 117 | 11.1 | 88.0 | 0.9 |

N: Number of participants with available results; % percentage of participants in each category; Below/Within/Above: values below/within/above normal laboratory ranges.

Note:	Difference to 100% represents percentage of participants with unknown value for the specified time point and laboratory parameter.

None of the laboratory abnormalities was considered serious or related to study vaccination.

**Supplementary table S4.** Distribution of haematology with respect to normal laboratory ranges (total vaccinated cohort)

|  |  |  | LD HA/AS03B | | | |  | LD HA/AS03A | | | |  | MD HA/AS03B | | | |  | MD HA/AS03A | | | |  | HD HA unadj | | | |  | Placebo | | | |
| --- | --- | --- | --- | --- | --- | --- | --- | --- | --- | --- | --- | --- | --- | --- | --- | --- | --- | --- | --- | --- | --- | --- | --- | --- | --- | --- | --- | --- | --- | --- | --- |
|  |  |  |  | Below | Within | Above |  |  | Below | Within | Above |  |  | Below | Within | Above |  |  | Below | Within | Above |  |  | Below | Within | Above |  |  | Below | Within | Above |
| Laboratory parameter | Timing |  | N | % | % | % |  | N | % | % | % |  | N | % | % | % |  | N | % | % | % |  | N | % | % | % |  | N | % | % | % |
| Basophils | Pre-vac. |  | 60 | 0.0 | 100.0 | 0.0 |  | 60 | 0.0 | 98.3 | 0.0 |  | 60 | 0.0 | 100.0 | 0.0 |  | 60 | 0.0 | 100.0 | 0.0 |  | 60 | 0.0 | 100.0 | 0.0 |  | 120 | 0.0 | 99.2 | 0.0 |
|  | Day 7 |  | 59 | 0.0 | 100.0 | 0.0 |  | 60 | 0.0 | 98.3 | 0.0 |  | 60 | 0.0 | 100.0 | 0.0 |  | 59 | 0.0 | 100.0 | 0.0 |  | 59 | 0.0 | 100.0 | 0.0 |  | 118 | 0.0 | 100.0 | 0.0 |
|  | Day 21 |  | 60 | 0.0 | 100.0 | 0.0 |  | 60 | 0.0 | 98.3 | 0.0 |  | 60 | 0.0 | 98.3 | 0.0 |  | 60 | 0.0 | 100.0 | 0.0 |  | 58 | 0.0 | 100.0 | 0.0 |  | 117 | 0.0 | 100.0 | 0.0 |
|  | Day 28 |  | 60 | 0.0 | 100.0 | 0.0 |  | 59 | 0.0 | 98.3 | 0.0 |  | 60 | 0.0 | 100.0 | 0.0 |  | 59 | 0.0 | 96.6 | 0.0 |  | 58 | 0.0 | 100.0 | 0.0 |  | 117 | 0.0 | 100.0 | 0.0 |
|  | Day 42 |  | 58 | 0.0 | 100.0 | 0.0 |  | 59 | 0.0 | 100.0 | 0.0 |  | 59 | 0.0 | 100.0 | 0.0 |  | 59 | 0.0 | 98.3 | 0.0 |  | 58 | 0.0 | 100.0 | 0.0 |  | 117 | 0.0 | 100.0 | 0.0 |
| Eosinophils | Pre-vac. |  | 60 | 15.0 | 81.7 | 3.3 |  | 60 | 11.7 | 86.7 | 0.0 |  | 60 | 15.0 | 83.3 | 1.7 |  | 60 | 10.0 | 86.7 | 3.3 |  | 60 | 8.3 | 91.7 | 0.0 |  | 120 | 8.3 | 90.8 | 0.0 |
|  | Day 7 |  | 59 | 8.5 | 86.4 | 5.1 |  | 60 | 8.3 | 86.7 | 3.3 |  | 60 | 8.3 | 90.0 | 1.7 |  | 59 | 3.4 | 94.9 | 1.7 |  | 59 | 13.6 | 86.4 | 0.0 |  | 118 | 9.3 | 89.0 | 1.7 |
|  | Day 21 |  | 60 | 10.0 | 81.7 | 8.3 |  | 60 | 5.0 | 90.0 | 3.3 |  | 60 | 5.0 | 91.7 | 1.7 |  | 60 | 3.3 | 96.7 | 0.0 |  | 58 | 13.8 | 86.2 | 0.0 |  | 117 | 9.4 | 88.9 | 1.7 |
|  | Day 28 |  | 60 | 5.0 | 90.0 | 5.0 |  | 59 | 8.5 | 86.4 | 3.4 |  | 60 | 8.3 | 90.0 | 1.7 |  | 59 | 8.5 | 88.1 | 0.0 |  | 58 | 5.2 | 93.1 | 1.7 |  | 117 | 12.0 | 86.3 | 1.7 |
|  | Day 42 |  | 58 | 8.6 | 86.2 | 5.2 |  | 59 | 5.1 | 91.5 | 3.4 |  | 59 | 10.2 | 86.4 | 3.4 |  | 59 | 11.9 | 86.4 | 0.0 |  | 58 | 0.0 | 100.0 | 0.0 |  | 117 | 8.5 | 88.9 | 2.6 |
| Hematocrit | Pre-vac. |  | 60 | 5.0 | 90.0 | 5.0 |  | 60 | 0.0 | 86.7 | 11.7 |  | 60 | 11.7 | 85.0 | 3.3 |  | 60 | 1.7 | 96.7 | 1.7 |  | 60 | 3.3 | 88.3 | 8.3 |  | 120 | 5.8 | 90.8 | 3.3 |
|  | Day 7 |  | 59 | 6.8 | 83.1 | 10.2 |  | 60 | 0.0 | 91.7 | 6.7 |  | 60 | 11.7 | 86.7 | 1.7 |  | 59 | 10.2 | 89.8 | 0.0 |  | 59 | 6.8 | 88.1 | 5.1 |  | 118 | 3.4 | 94.9 | 1.7 |
|  | Day 21 |  | 60 | 3.3 | 91.7 | 5.0 |  | 60 | 1.7 | 93.3 | 3.3 |  | 60 | 11.7 | 81.7 | 5.0 |  | 60 | 5.0 | 93.3 | 1.7 |  | 58 | 5.2 | 93.1 | 1.7 |  | 117 | 6.0 | 92.3 | 1.7 |
|  | Day 28 |  | 60 | 5.0 | 93.3 | 1.7 |  | 59 | 5.1 | 89.8 | 3.4 |  | 60 | 8.3 | 90.0 | 1.7 |  | 59 | 6.8 | 88.1 | 1.7 |  | 58 | 5.2 | 93.1 | 1.7 |  | 117 | 9.4 | 88.9 | 1.7 |
|  | Day 42 |  | 58 | 6.9 | 84.5 | 8.6 |  | 59 | 1.7 | 94.9 | 3.4 |  | 59 | 10.2 | 86.4 | 3.4 |  | 59 | 3.4 | 93.2 | 1.7 |  | 58 | 3.4 | 93.1 | 3.4 |  | 117 | 4.3 | 93.2 | 2.6 |
| Hemoglobin | Pre-vac. |  | 60 | 10.0 | 88.3 | 1.7 |  | 60 | 5.0 | 86.7 | 6.7 |  | 60 | 13.3 | 85.0 | 1.7 |  | 60 | 15.0 | 85.0 | 0.0 |  | 60 | 6.7 | 90.0 | 3.3 |  | 120 | 10.0 | 89.2 | 0.8 |
|  | Day 7 |  | 59 | 10.2 | 89.8 | 0.0 |  | 60 | 6.7 | 91.7 | 0.0 |  | 60 | 18.3 | 80.0 | 1.7 |  | 59 | 18.6 | 81.4 | 0.0 |  | 59 | 11.9 | 86.4 | 1.7 |  | 118 | 12.7 | 87.3 | 0.0 |
|  | Day 21 |  | 60 | 11.7 | 86.7 | 1.7 |  | 60 | 8.3 | 88.3 | 1.7 |  | 60 | 16.7 | 78.3 | 3.3 |  | 60 | 13.3 | 86.7 | 0.0 |  | 58 | 8.6 | 91.4 | 0.0 |  | 117 | 16.2 | 83.8 | 0.0 |
|  | Day 28 |  | 60 | 8.3 | 91.7 | 0.0 |  | 59 | 11.9 | 84.7 | 1.7 |  | 60 | 15.0 | 83.3 | 1.7 |  | 59 | 16.9 | 78.0 | 1.7 |  | 58 | 6.9 | 93.1 | 0.0 |  | 117 | 13.7 | 86.3 | 0.0 |
|  | Day 42 |  | 58 | 10.3 | 86.2 | 3.4 |  | 59 | 11.9 | 86.4 | 1.7 |  | 59 | 13.6 | 84.7 | 1.7 |  | 59 | 11.9 | 84.7 | 1.7 |  | 58 | 8.6 | 91.4 | 0.0 |  | 117 | 12.0 | 88.0 | 0.0 |
| Lymphocytes | Pre-vac. |  | 60 | 0.0 | 100.0 | 0.0 |  | 60 | 1.7 | 96.7 | 0.0 |  | 60 | 1.7 | 96.7 | 1.7 |  | 60 | 0.0 | 98.3 | 1.7 |  | 60 | 0.0 | 100.0 | 0.0 |  | 120 | 1.7 | 97.5 | 0.0 |
|  | Day 7 |  | 59 | 1.7 | 98.3 | 0.0 |  | 60 | 0.0 | 98.3 | 0.0 |  | 60 | 1.7 | 98.3 | 0.0 |  | 59 | 0.0 | 98.3 | 1.7 |  | 59 | 0.0 | 100.0 | 0.0 |  | 118 | 0.8 | 99.2 | 0.0 |
|  | Day 21 |  | 60 | 0.0 | 100.0 | 0.0 |  | 60 | 1.7 | 93.3 | 3.3 |  | 60 | 3.3 | 95.0 | 0.0 |  | 60 | 1.7 | 96.7 | 1.7 |  | 58 | 1.7 | 98.3 | 0.0 |  | 117 | 0.9 | 99.1 | 0.0 |
|  | Day 28 |  | 60 | 0.0 | 100.0 | 0.0 |  | 59 | 0.0 | 96.6 | 1.7 |  | 60 | 1.7 | 98.3 | 0.0 |  | 59 | 1.7 | 93.2 | 1.7 |  | 58 | 0.0 | 100.0 | 0.0 |  | 117 | 0.0 | 100.0 | 0.0 |
|  | Day 42 |  | 58 | 0.0 | 100.0 | 0.0 |  | 59 | 1.7 | 96.6 | 1.7 |  | 59 | 0.0 | 100.0 | 0.0 |  | 59 | 1.7 | 94.9 | 1.7 |  | 58 | 0.0 | 100.0 | 0.0 |  | 117 | 0.0 | 100.0 | 0.0 |
| Monocytes | Pre-vac. |  | 60 | 11.7 | 88.3 | 0.0 |  | 60 | 13.3 | 85.0 | 0.0 |  | 60 | 11.7 | 88.3 | 0.0 |  | 60 | 8.3 | 91.7 | 0.0 |  | 60 | 15.0 | 85.0 | 0.0 |  | 120 | 11.7 | 87.5 | 0.0 |
|  | Day 7 |  | 59 | 15.3 | 84.7 | 0.0 |  | 60 | 15.0 | 83.3 | 0.0 |  | 60 | 6.7 | 93.3 | 0.0 |  | 59 | 5.1 | 94.9 | 0.0 |  | 59 | 15.3 | 84.7 | 0.0 |  | 118 | 13.6 | 86.4 | 0.0 |
|  | Day 21 |  | 60 | 11.7 | 88.3 | 0.0 |  | 60 | 13.3 | 85.0 | 0.0 |  | 60 | 10.0 | 88.3 | 0.0 |  | 60 | 8.3 | 91.7 | 0.0 |  | 58 | 13.8 | 86.2 | 0.0 |  | 117 | 12.0 | 88.0 | 0.0 |
|  | Day 28 |  | 60 | 10.0 | 90.0 | 0.0 |  | 59 | 13.6 | 84.7 | 0.0 |  | 60 | 13.3 | 85.0 | 1.7 |  | 59 | 5.1 | 91.5 | 0.0 |  | 58 | 12.1 | 87.9 | 0.0 |  | 117 | 14.5 | 85.5 | 0.0 |
|  | Day 42 |  | 58 | 8.6 | 91.4 | 0.0 |  | 59 | 11.9 | 86.4 | 1.7 |  | 59 | 10.2 | 89.8 | 0.0 |  | 59 | 6.8 | 91.5 | 0.0 |  | 58 | 5.2 | 94.8 | 0.0 |  | 117 | 17.1 | 82.9 | 0.0 |
| Neutrophils | Pre-vac. |  | 60 | 3.3 | 90.0 | 6.7 |  | 60 | 3.3 | 90.0 | 5.0 |  | 60 | 1.7 | 88.3 | 10.0 |  | 60 | 1.7 | 86.7 | 11.7 |  | 60 | 1.7 | 96.7 | 1.7 |  | 120 | 0.8 | 95.8 | 2.5 |
|  | Day 7 |  | 59 | 3.4 | 94.9 | 1.7 |  | 60 | 3.3 | 93.3 | 1.7 |  | 60 | 5.0 | 90.0 | 5.0 |  | 59 | 1.7 | 93.2 | 5.1 |  | 59 | 0.0 | 94.9 | 5.1 |  | 118 | 1.7 | 94.1 | 4.2 |
|  | Day 21 |  | 60 | 3.3 | 95.0 | 1.7 |  | 60 | 3.3 | 93.3 | 1.7 |  | 60 | 5.0 | 88.3 | 5.0 |  | 60 | 3.3 | 95.0 | 1.7 |  | 58 | 0.0 | 98.3 | 1.7 |  | 117 | 3.4 | 89.7 | 6.8 |
|  | Day 28 |  | 60 | 5.0 | 88.3 | 6.7 |  | 59 | 5.1 | 89.8 | 3.4 |  | 60 | 5.0 | 93.3 | 1.7 |  | 59 | 1.7 | 91.5 | 3.4 |  | 58 | 5.2 | 94.8 | 0.0 |  | 117 | 3.4 | 93.2 | 3.4 |
|  | Day 42 |  | 58 | 3.4 | 93.1 | 3.4 |  | 59 | **6.8*** | 88.1 | 5.1 |  | 59 | 5.1 | 91.5 | 3.4 |  | 59 | 6.8 | 91.5 | 0.0 |  | 58 | 5.2 | 94.8 | 0.0 |  | 117 | 4.3 | 92.3 | 3.4 |
| Platelets | Pre-vac. |  | 60 | 0.0 | 98.3 | 1.7 |  | 60 | 1.7 | 95.0 | 1.7 |  | 60 | 1.7 | 93.3 | 5.0 |  | 60 | 1.7 | 93.3 | 5.0 |  | 60 | 0.0 | 98.3 | 1.7 |  | 120 | 0.0 | 100.0 | 0.0 |
|  | Day 7 |  | 59 | 0.0 | 98.3 | 1.7 |  | 60 | 0.0 | 98.3 | 0.0 |  | 60 | 0.0 | 98.3 | 1.7 |  | 59 | 0.0 | 91.5 | 6.8 |  | 59 | 0.0 | 96.6 | 3.4 |  | 118 | 0.0 | 100.0 | 0.0 |
|  | Day 21 |  | 60 | 0.0 | 98.3 | 1.7 |  | 60 | 1.7 | 96.7 | 0.0 |  | 60 | 1.7 | 93.3 | 3.3 |  | 60 | 0.0 | 98.3 | 1.7 |  | 58 | 0.0 | 98.3 | 1.7 |  | 117 | 0.9 | 99.1 | 0.0 |
|  | Day 28 |  | 60 | 0.0 | 98.3 | 1.7 |  | 59 | 1.7 | 96.6 | 0.0 |  | 60 | 0.0 | 95.0 | 5.0 |  | 59 | 0.0 | 93.2 | 3.4 |  | 58 | 0.0 | 94.8 | 5.2 |  | 117 | 0.0 | 99.1 | 0.9 |
|  | Day 42 |  | 58 | 0.0 | 100.0 | 0.0 |  | 59 | 0.0 | 100.0 | 0.0 |  | 59 | 1.7 | 96.6 | 1.7 |  | 59 | 0.0 | 93.2 | 5.1 |  | 58 | 0.0 | 96.6 | 3.4 |  | 117 | 0.0 | 96.6 | 3.4 |
| Erythrocytes | Pre-vac. |  | 60 | 3.3 | 96.7 | 0.0 |  | 60 | 0.0 | 93.3 | 5.0 |  | 60 | 5.0 | 93.3 | 1.7 |  | 60 | 5.0 | 95.0 | 0.0 |  | 60 | 3.3 | 93.3 | 3.3 |  | 120 | 8.3 | 89.2 | 2.5 |
|  | Day 7 |  | 59 | 5.1 | 94.9 | 0.0 |  | 60 | 1.7 | 95.0 | 1.7 |  | 60 | 6.7 | 91.7 | 1.7 |  | 59 | 10.2 | 89.8 | 0.0 |  | 59 | 3.4 | 96.6 | 0.0 |  | 118 | 6.8 | 89.0 | 4.2 |
|  | Day 21 |  | 60 | 1.7 | 96.7 | 1.7 |  | 60 | 1.7 | 93.3 | 3.3 |  | 60 | 3.3 | 93.3 | 1.7 |  | 60 | 6.7 | 91.7 | 1.7 |  | 58 | 3.4 | 94.8 | 1.7 |  | 117 | 6.0 | 90.6 | 3.4 |
|  | Day 28 |  | 60 | 6.7 | 93.3 | 0.0 |  | 59 | 5.1 | 93.2 | 0.0 |  | 60 | 5.0 | 93.3 | 1.7 |  | 59 | 5.1 | 89.8 | 1.7 |  | 58 | 3.4 | 94.8 | 1.7 |  | 117 | 7.7 | 90.6 | 1.7 |
|  | Day 42 |  | 58 | 3.4 | 96.6 | 0.0 |  | 59 | 0.0 | 98.3 | 1.7 |  | 59 | 5.1 | 94.9 | 0.0 |  | 59 | 3.4 | 93.2 | 1.7 |  | 58 | 3.4 | 96.6 | 0.0 |  | 117 | 6.0 | 89.7 | 4.3 |
| Leukocytes | Pre-vac. |  | 60 | 3.3 | 90.0 | 6.7 |  | 60 | 1.7 | 85.0 | 11.7 |  | 60 | 3.3 | 88.3 | 8.3 |  | 60 | 1.7 | 90.0 | 8.3 |  | 60 | 1.7 | 96.7 | 1.7 |  | 120 | 0.8 | 93.3 | 5.0 |
|  | Day 7 |  | 59 | 3.4 | 94.9 | 1.7 |  | 60 | 1.7 | 93.3 | 3.3 |  | 60 | 3.3 | 91.7 | 5.0 |  | 59 | 3.4 | 89.8 | 6.8 |  | 59 | 1.7 | 91.5 | 6.8 |  | 118 | 2.5 | 93.2 | 4.2 |
|  | Day 21 |  | 60 | 5.0 | 93.3 | 1.7 |  | 60 | 1.7 | 93.3 | 3.3 |  | 60 | 5.0 | 85.0 | 8.3 |  | 60 | 1.7 | 96.7 | 1.7 |  | 58 | 1.7 | 96.6 | 1.7 |  | 117 | 1.7 | 93.2 | 5.1 |
|  | Day 28 |  | 60 | 0.0 | 93.3 | 6.7 |  | 59 | 1.7 | 88.1 | 8.5 |  | 60 | 5.0 | 91.7 | 3.3 |  | 59 | 1.7 | 89.8 | 5.1 |  | 58 | 3.4 | 94.8 | 1.7 |  | 117 | 4.3 | 94.0 | 1.7 |
|  | Day 42 |  | 58 | 6.9 | 89.7 | 3.4 |  | 59 | 3.4 | 88.1 | 8.5 |  | 59 | 5.1 | 91.5 | 3.4 |  | 59 | 3.4 | 93.2 | 1.7 |  | 58 | 1.7 | 96.6 | 1.7 |  | 117 | 1.7 | 92.3 | 6.0 |

* One Grade 3 decreased neutrophil count was reported for one study participant at this timepoint in the LD HA/AS03A group. Previous neutrophil levels were within normal range. The individual was on oral treatment with citralipram and omeprazole for depression and heartburn at the Day 42 visit.

N: Number of participants with available results; % percentage of participants in each category; Below/Within/Above: values below/within/above normal laboratory ranges

Note:	Difference to 100% represents percentage of participants with unknown value for the specified time point and laboratory parameter.

None of the laboratory abnormalities was considered serious or related to study vaccination.

**Supplementary figure S1.** Seropositivity rate and GMTs for vaccine-homologous HI antibodies (A) and for vaccine-heterologous HI antibodies (B) (adapted per protocol cohort)

| **A** |  |
| --- | --- |
| **B** |  |

CI: confidence interval; D: day; GMT: geometric mean titer; HA: hemagglutinin; HD: high dose; HI: haemagglutination inhibition; LD: low dose; MD: medium dose; PRE: prevaccination; D21: 21 days after first vaccination; D42: 21 days after second vaccination; D182: 182 days after the first vaccination; D385: 385 days after the first vaccination

Bars show the GMT values; Error bars show the 95% CI; Values above the bars show the seropositivity rate (%); Horizontal gridline shows the cut-off value of the assay. The adapted per protocol cohort includes Day 0, Day 21 and Day 42 data from the Day 42 per protocol cohort; Day 182 data from the Day 182 per protocol cohort, and Day 385 data from the Day 385 per protocol cohort.
